# Efficacy of Ginger Supplementation in Relieving Persistent Hypothyroid Symptoms in Patients with Controlled Primary Hypothyroidism: A Pilot Randomized, Double-Blind, Placebo-Controlled Clinical Trial

**DOI:** 10.1155/2022/5456855

**Published:** 2022-01-20

**Authors:** Hamide Ashraf, Mojtaba Heydari, Mesbah Shams, Mohammad Mehdi Zarshenas, Ali Tavakoli, Mehrab Sayadi

**Affiliations:** ^1^Department of Persian Medicine, School of Medicine, Shiraz University of Medical Science, Shiraz, Iran; ^2^Research Center for Traditional Medicine and History of Medicine, Shiraz University of Medical Sciences, Shiraz, Iran; ^3^Endocrinology and Metabolism Research Center, Shiraz University of Medical Science, Shiraz, Iran; ^4^Medicinal Plants Processing Research Center, School of Pharmacy, Shiraz University of Medical Science, Shiraz, Iran; ^5^Department of Phytopharmaceuticals (Traditional Pharmacy), School of Pharmacy, Shiraz University of Medical Science, Shiraz, Iran; ^6^Epilepsy Research Center, Shiraz University of Medical Sciences, Shiraz, Iran; ^7^Cardiovascular Research Center, Shiraz University of Medical Sciences, Shiraz, Iran

## Abstract

Primary hypothyroidism is a common disease. Some patients have persistent symptoms despite normal serum thyroid-stimulating hormone (TSH) levels. Ginger is reported to be beneficial in relieving similar symptoms. Our aim was to evaluate the efficacy of ginger supplementation in relieving persistent symptoms in these patients. In this randomized, double-blind, placebo-controlled clinical trial, 60 hypothyroid patients aged 20–60 years with normal serum TSH concentrations were randomly allocated to two equal parallel study groups of ginger (500 mg twice a day) or placebo for 30 days. Hypothyroid symptoms were evaluated as the primary outcome using the Thyroid Symptom Rating Questionnaire (ThySRQ) before and after the intervention. Anthropometric measures and laboratory indices including TSH, triglyceride (TG), total cholesterol (TChol), and fasting blood sugar (FBS) were considered as secondary outcomes. A significant lower mean total ThySRQ score (8.63 ± 5.47 vs. 15.76 ± 6.09, *P* < 0.001) was observed in the ginger group compared to the control group. Ginger led to significant improvements in the mean scores of the weight gain, cold intolerance, constipation, dry skin, appetite, memory loss, concentration disturbance, and feeling giddy or dizzy domains (*P* < 0.001). However, no significant improvements were observed in hair loss, nail fragility, hearing, hoarseness, speech, and depression or feeling down (*P* > 0.05). Ginger supplementation also led to a significant decrease in body weight, body mass index, waist circumference, serum TSH, FBS, TG, and TChol levels compared to the placebo. In summary according to preliminary results of this study, ginger supplementation can help relieve persistent hypothyroid symptoms. Also, it may have beneficial effects in terms of weight reduction and regulation of the FBS and lipid profile in hypothyroid patients.

## 1. Introduction

Primary hypothyroidism is a clinical syndrome in which the thyroid gland does not produce enough thyroid hormones. The disease is classified as overt hypothyroidism and subclinical hypothyroidism [1, 2]. Overt hypothyroidism is characterized by an increase in the serum level of thyroid-stimulating hormone (TSH) and a decrease in the serum level of free thyroxine (T4), whereas the main feature of subclinical hypothyroidism is a normal free T4 concentration in the presence of an elevated serum TSH concentration [3, 4]. The prevalence of hypothyroidism is generally 1-2%, with five to eight times greater prevalence among women [5]. The most common symptoms and signs of hypothyroidism include fatigue, voice changes, cold intolerance, weight gain, constipation, dry skin, malaise, menstrual irregularities, decreased resting metabolism, dyslipidemia, and insulin resistance [6, 7].

Replacement synthetic thyroxine (levothyroxine) is the standard therapy for hypothyroidism [8, 9]. The goal of therapy is the normalization of the serum TSH level [8, 9]. After thyroid hormone replacement therapy, approximately 15% of patients remain symptomatic despite achieving normal serum TSH levels [6, 10]. The pathophysiology of persistent hypothyroid symptoms despite adequate hormone replacement therapy is not well understood [11, 12], and no definitive treatment is available [13, 14]. Levothyroxine-liothyronine combination therapy is a suggested treatment for this group of patients with conflicting results [13, 15].

*Zingiber officinale* is a perennial plant from the Zingiberaceae family. Its rhizome (underground stem), commonly known as ginger, is widely used as a popular spice in different cultures around the world [16, 17]. The Food and Drug Administration (FDA) recognizes ginger as a safe dietary supplement [18]. It also has long been used in traditional medicines as a hot remedy for the treatment of cold temperament symptoms and signs such as tiredness, constipation, obesity, and menstrual irregularities [19, 20]. According to the similarities between the mentioned symptoms and hypothyroid symptoms, warm remedies such as ginger are recommended by traditional medicine practitioners for these patients [20]. Besides, several scientific investigations have confirmed the therapeutic effects of ginger on hyperlipidemia [21, 22], insulin resistance [23, 24], and obesity [22, 25], which are all common comorbidities in hypothyroid patients. Recent studies also have shown the protective effect of ginger against thyroid damage in animals and humans [16, 26, 27].

Based on the traditional use of ginger supplementation in the improvement of hypothyroidism-like symptoms as well as current evidence of the therapeutic effects of ginger on the comorbidities of hypothyroidism, the present study was designed to evaluate the efficacy of ginger supplementation in alleviating persistent hypothyroid symptoms and treating the related comorbidities among patients with adequate hormone replacement therapy.

## 2. Materials and Methods

### 2.1. Registration and Ethical Issues

The current study was registered in the Iranian Registry of Clinical Trials (IRCT; “this trial is registered with https://trialsearch.who.int/?TrialID=IRCT20191231045961N1”). All participants provided signed informed consent. The study protocol was approved by the Ethics Committee of Shiraz University of Medical Sciences (IR.SUMS.MED.REC.1398.548).

### 2.2. Study Design

This study was a single-center, double-blind, placebo-controlled, randomized clinical trial with two parallel arms. No changes were made to the method after starting the trial.

### 2.3. Participants

Participants enrolled in this study were men and women aged 20–60 years with a previously confirmed diagnosis of overt primary hypothyroidism under hormone replacement therapy with normal serum TSH concentration (0.3–4.5 mIU/L) and a body mass index (BMI) of 19–35 kg/m^2^ who were referred to Yasuj (south of Iran) endocrinology clinics between August and November 2020.

Exclusion criteria were pregnancy, lactation, alcohol consumption, diabetes mellitus, dyslipidemia, vitamin D deficiency, co-occurrence of other thyroid diseases, malignancy, liver and kidney failure, hypopituitarism, malabsorption disorders (celiac disease, inflammatory bowel disease, and atrophic gastritis), concomitant use of drugs such as multivitamins, minerals, phenobarbital, phenytoin, carbamazepine, rifampin, glucocorticoids, warfarin, statins, estrogen, and raloxifene, and history of hypersensitivity to ginger supplements.

### 2.4. Sample Size and Randomization

By applying data of previous studies [16] and using the MedCalc software with a 5% significance level and a power of 80%, the sample size in each group was determined to be 30 cases. Based on the list from the random allocation software, thirty patients were placed in each group using the block randomization method with a block size of 4.

### 2.5. Ginger and Placebo Capsules' Preparation

Ginger rhizomes were purchased from the local market and authenticated by a botanist from the Department of Phytopharmaceuticals, School of Pharmacy, Shiraz University of Medical Sciences, Shiraz, Iran, with a specified voucher number (PM -1142). Then, the ginger rhizomes were pulverized and exposed to ultraviolet light. Then, capsules were filled with 500 mg of ginger powder. To prepare the placebo, the second group of capsules were filled with 500 mg of roasted starch. To ensure odor matching, the placebo capsules (with the same appearance as ginger capsules) were placed in the presence of ginger powder for two weeks. Then, both groups of capsules were packed using the same packaging but were coded with different codes. All of these steps were performed in the Department of Traditional Pharmacy of the Faculty of Pharmacy of Shiraz University of Medical Sciences. To keep the study double-blind, the codes were broken after recording the information of the last participant.

### 2.6. Intervention

Prior to commencing the intervention, the objectives of the study and the possible side effects of the drugs were explained to each patient, and written informed consent was obtained. Then, study questionnaires were filled out by the enrolled patients to assess their symptoms. Next, the patients were randomly assigned to the two study groups. Finally, according to previous studies [28–30], the participants received one 500 mg capsule containing ginger powder in the intervention group and one starch-containing capsule in the placebo group 30 min before lunch and dinner (twice daily) for 30 days. All participants were on medication (levothyroxine 30 min before breakfast) and were advised not to change their levothyroxine dose, lifestyle, and physical activity during the study.

### 2.7. Outcome Measures

The primary outcome was the clinical symptoms as evaluated by the Thyroid Symptom Rating Questionnaire (ThySRQ), which is a validated disease-specific questionnaire for hypothyroidism [31]. The ThySRQ consists of 15 questions with a four-point symptom bother scale (not at all (0), a little (1), quite a bit (2), and very much (3)) that asks about the symptoms of hypothyroidism that patients may have experienced in recent weeks (3–6 weeks) and measures the perceived symptom severity. Secondary outcome measures were body weight, BMI, waist circumference, triglyceride (TG), total cholesterol (TChol), fasting blood sugar (FBS), and TSH, which were evaluated before and after the intervention. Blood samples were taken from all participants after 14 hours of overnight fasting. All patients were instructed to report any observed adverse events during the study.

### 2.8. Statistical Analysis

All statistical analyses were performed using the SPSS software (version 21.0; IBM Corp., Armonk, NY, USA). The normality of the data was examined using the Shapiro–Wilk test. The independent sample *t*-test and paired sample *t*-test were used to compare quantitative variables. The chi-squared test was used to compare qualitative variables in both groups. If the chi-square test was not suitable, Fisher's exact test was used. If the data distribution was not normal, the median and midrange were used to describe the data; such data were assessed using the Mann–Whitney *U* test for intergroup comparison and the Wilcoxon test for intragroup comparison. Quantitative data were expressed as mean ± standard deviation (SD), while qualitative variables were reported using frequency and percentage. In all tests, statistical significance was considered when the *P* value was below 0.05.

## 3. Results

### 3.1. Study Flow

**Figure 1:**
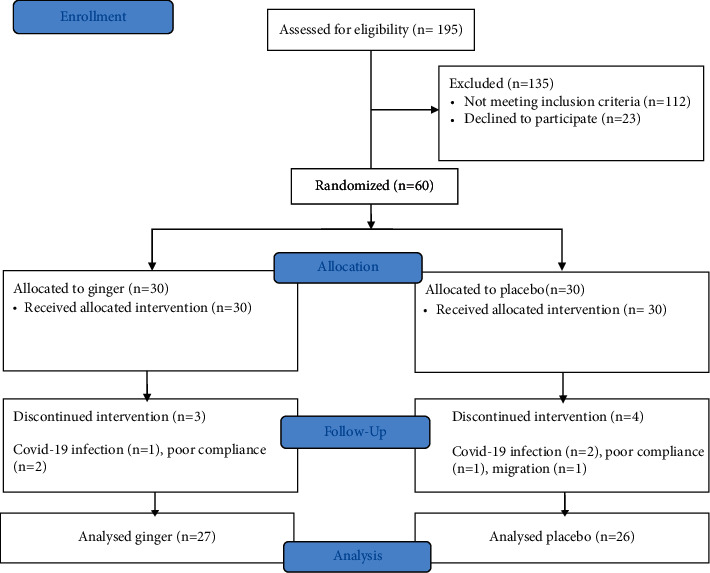
CONSORT flow diagram of the patients' enrollment and follow-up.

In the course of the study (30 days), out of the 60 patients with a mean age of 40.47 ± 8.35 years and a mean weight of 83.45 ± 16.93 kg who were enrolled, 7 patients were excluded because of coronavirus disease 2019 (COVID-19) affliction, poor compliance, or migration. Finally, 53 patients reached the data analysis stage, of which 27 patients were in the ginger group and 26 were in the placebo group. The details of patients' enrollment, follow-up, and analysis are demonstrated in Figure 1.

### 3.2. Baseline Demographic and Anthropometric Characteristics

The baseline demographic and anthropometric characteristics of the patients are shown in Table 1. Comparison of these characteristics shows that there was no significant difference in age, sex, BMI, and waist circumference between the study groups (*P* > 0.05).

### 3.3. Hypothyroid Symptoms

**Figure 2:**
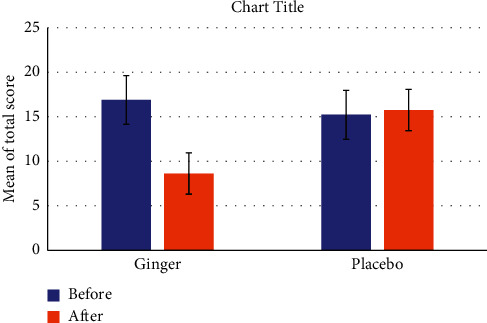
Change of total ThySRQ score between and within groups before and after the intervention.

After the patients receiving ginger supplementation showed a significant decrease in the mean total ThySRQ score compared to the placebo group (Figure 2). Specifically, ginger led to significant changes in weight gain, cold feeling, constipation, dry skin, appetite, memory loss, concentration disturbance, and feeling giddy or dizzy domains, while the remaining domains showed no significant changes (*P* > 0.05). The details of information on the mean ThySRQ score before and after the intervention in both study groups are summarized in Table 2.

### 3.4. Anthropometric Measures

Ginger supplementation led to a significant decrease in body weight, BMI, and waist circumference when compared to the placebo group. The anthropometric outcomes before and after the intervention are summarized in Table 3.

### 3.5. Laboratory Indices

Clinically minor but statistically significant improvements were also observed in the laboratory outcomes including the TSH, FBS, TG, and TChol levels in the ginger group compared to the control. The related data are presented in Table 3.

### 3.6. Physical Activity

The participants' level of physical activity according to the International Physical Activity Questionnaire (IPAQ) is reported in Table 3. The results indicate that there was no significant difference within and between the groups in terms of physical activity at the beginning and after the intervention.

### 3.7. Adverse Events

No patient in the ginger or placebo group reported any adverse event throughout the study period.

### 3.8. Gender-Based Subgroup Analysis

Among the total 53 patients included in the final analysis, 40 patients (75.5%) were female. There was no statistically significant difference between the mean scores of ThySRQ domains and laboratory indices (TSH, FBS, TG, and TChol) in males and females receiving ginger (*P* > 0.05). The results of gender-based subgroup analysis are presented in Table 4.

## 4. Discussion

The present study results revealed that ginger supplementation in the dose of 1000 mg/d can alleviate persistent hypothyroid symptoms after adequate hormone replacement therapy. It also showed beneficial effects on certain anthropometric and laboratory outcomes in these patients.

Hypothyroidism is a chronic disease with multiple physiological and psychological symptoms that require long-life treatment, exerting a remarkable negative effect on the patient's quality of life (QoL) [32]. Winther KH et al. demonstrated that treatment with levothyroxine for six weeks significantly improved the QoL of hypothyroidism patients compared to the general population [33]. Nevertheless, approximately 15% of patients remain symptomatic despite achieving normal serum TSH levels [34]. Symptoms of hypothyroidism include a wide range such as fatigue, cold intolerance, dry skin, hair loss, constipation, decreased appetite, memory loss, concentration disturbance, and feeling giddy or dizzy which are sometimes accompanied by other underlying conditions, including chronic depression, gastrointestinal problems, or anemia [3, 35]. Therefore, questionnaires which rate thyroid symptoms such as ThySRQ are nonspecific, and the recorded symptoms can be due to a wide range of diseases.

In our study, before the intervention, the most common persistent symptoms despite adequate thyroid hormone replacement were cold intolerance and tiredness according to ThySRQ, which is in concordance with the studies of Winther et al. [33] and McMillan et al. [31]. MacLean et al. also suggested that tiredness, weight gain, and feeling cold are the most common symptoms in euthyroid yet symptomatic patients [36]. There is currently no definitive treatment to control symptoms in symptomatic patients who are biochemically euthyroid [12]. In our study, some symptoms were ameliorated significantly in the ginger group compared with the placebo group according to ThySRQ. These symptoms included weight gain, cold intolerance, constipation, dry skin, decreased appetite, memory loss, concentration disturbance, and feeling giddy or dizzy.

There is limited human and animal evidence about the effects of ginger supplementation on thyroid gland function. Mohammed et al. demonstrated that ginger extract had an antioxidant and protective effect against bisphenol A-induced thyroid injury in male rats, acting through the nuclear factor-like 2 (Nrf2)/heme oxygenase-1 (HO-1) pathway [26]. Their findings are in accordance with the results of Al-Amoudi et al. [27], which showed the ameliorative effect of the ginger extract on thyroid toxicity induced by lambda-cyhalothrin in albino rats. In these studies, intragastric gavage of bisphenol A or lambda-cyhalothrin created a cascade of histological changes in the thyroid gland, which led to decreased serum levels of thyroid hormones and increased TSH concentration. Notably, the synchronic gavage of the ginger extract resulted in a protective effect against the histological changes and led to a significant increase in the serum levels of thyroid hormones together with a remarkable decrease in TSH concentration [26,27]. In a clinical trial recently published by Mahassni et al., a result similar to that of our study was observed about the effect of ginger supplementation on the TSH level in patients with hypothyroidism. However, this group of researchers administered a higher dose of ginger (8.3 g of ginger extract) for a shorter study duration (3 weeks) [16]. Another study performed by Barari et al. demonstrated that the consumption of 10 mg/kg/day ginger extract for six weeks had no significant effect on TSH concentration, contrasting with our findings [37]. However, that study included a healthy population, whereas all of our patients suffered from primary hypothyroidism.

Besides improved TSH, other laboratory indices including FBS, TG, and TChol also improved after ginger supplementation compared with the baseline and the placebo group. Previous studies have shown the antidiabetic and antilipidemic effects of the ginger extract in animals and humans [38, 39]. A study conducted by Al Hroob et al. in 2018 showed that the daily consumption of 800 mg/kg/day ginger extract for 6 weeks significantly reduced the FBS concentration in rats with streptozotocin-induced diabetes [40]. Chukwudike et al. also demonstrated the dose-dependent antidiabetic effect of ginger aqueous extract in male Wistar rats [41]. Subbaiah and colleagues revealed that the daily administration of 50–250 mg/kg ginger extract significantly reduced the lipid (TG and TChol) levels in male albino Wister rats [42]. In concordance with their study, Akanfe et al. explained that gavage of 400–600 mg/kg/day of ginger extract for four weeks decreased the TG and TChol concentration significantly in female Wistar rats [43]. Human studies also support these results. Murad et al. indicated that the daily consumption of 5 g of ginger powder for 12 weeks can reduce the TChol concentration in the healthy population [21]. Besides, Mushtaq et al. showed a significant decrease in the TChol concentration after 30 days of treatment with 3 g of ginger powder in patients with hyperlipidemia [44]. El Gayar et al. also demonstrated similar results in patients with diabetes mellitus after the administration of 1.8 g of ginger powder for 8 weeks, which led to reduced FBS, TG, and TChol concentrations [45]. However, no study before our report has evaluated such effects of ginger among patients with hypothyroidism.

Improvement of anthropometric measures in hypothyroid patients receiving ginger supplementation was another finding of our study. Although daily consumption of 1000 mg of ginger led to weight loss and body mass index, lack of measurement of daily calorie intake and potential nonreported concomitant use of weight reduction drugs by patients are confounding factors that can affect our results. Nevertheless, previous animal studies showed significant effects of ginger on obesity [46,47]. However, there is controversy about the beneficial effects of ginger on obesity in human studies [48,49], which may be due to differences in participants, duration, and the dose and pharmaceutical form of ginger. Similar to our study, Attari et al. illustrated that the administration of 2 g of ginger for 12 weeks in obese women significantly reduced the BMI and waist circumference compared with the baseline and the placebo group [17]. Also, Murad et al. demonstrated that the consumption of 5 g of ginger powder for 3 months significantly decreased the body weight in hyperlipidemic patients [21]. In a study conducted by Mozaffari et al. on 88 patients with type 2 diabetes, it was shown that the daily consumption of 3 g of ginger powder for 8 weeks did not affect the BMI [50]. Similarly, Arablou et al. explained that the administration of 1600 mg of ginger powder for 12 weeks in patients with type 2 diabetes had no effect on BMI and waist circumference [38]. Our results showed for the first time that ginger improved the anthropometric outcomes in patients with hypothyroidism.

The Food and Drug Administration (FDA) recognizes ginger as a safe dietary supplement [18]. Clinical studies have shown that consuming ginger up to 2 grams per day has the least toxicity to humans. The maximum dose of ginger is 6 grams per day [51]. No serious side effects of using this plant have been observed in the therapeutic dose range. In some studies, hypersensitivity reactions have been reported in the form of dermatitis [52]. In some cases, patients complained of dyspepsia and worsening of gastrointestinal symptoms [53]. In the present study, no serious side effects of ginger consumption were reported, which may be due to the low dose and short duration of consumption.

Besides the strength of using a prospective design in this clinical trial and comparing the use of the studied agent against a placebo, our study had some limitations. The small sample size, short duration of the intervention, use of only one dose of ginger, and lack of assessment of daily calorie intake were the most important limitations.

## 5. Conclusion

According to preliminary results of this study, it was demonstrated that the daily consumption of 1000 mg of ginger powder in patients with primary hypothyroidism on adequate hormone replacement (biochemically euthyroid) led to a significant reduction in hypothyroid symptoms. The most responsive symptoms to ginger supplementation were weight gain, cold intolerance, constipation, dry skin, decreased appetite, memory loss, concentration disturbance, and feeling giddy or dizzy. Furthermore, anthropometric outcomes including weight, BMI, and waist circumference as well as laboratory parameters such as FBS, TG, and total cholesterol also improved with ginger supplementation. It can be concluded that ginger supplementation in hypothyroid patients can help control persistent symptoms, decrease weight, and regulate the FBS and lipid profile. However, further human studies with larger sample sizes, longer durations, different ginger doses, and a follow-up period after discontinuation of the supplement are recommended.

## Figures and Tables

**Table 1:** Baseline demographic and anthropometric and laboratory characteristics of hypothyroid patients.

Characteristics	Ginger (mean ± SD), *n* = 27	Placebo (mean ± SD), *n* = 26	*P* value^†^
Gender (male/female)	9 (33%)/18 (67%)	4 (15%)/22 (85%)	0.129
Age (years)	42.44 ± 8.48	38.42 ± 7.86	0.08
BMI (kg/m^2^)	31.17 ± 5.28	31.29 ± 5.12	0.928
Waist size (cm)	110.3 ± 11.52	109.5 ± 13.92	0.821
TSH	3.62 ± 2.21	2.87 ± 1.88	0.185
IPAQ score	200.36 ± 95.69	200.35 ± 61.44	1.000

^**†**^ *t*-test for quantitative variable; chi-square for qualitative variable. BMI: body mass index; TSH: thyroid-stimulating hormone; IPAQ: International Physical Activity Questionnaire.

**Table 2:** Comparison of the changes in mean Hypothyroidism Symptom Rating Questionnaire (ThySRQ) scores between intervention groups.

ThySRQ domains/intervention		Ginger (mean ± SD), *n* = 27	Placebo (mean ± SD), *n* = 26	*P* value^*∗*^
Tiredness	Before	2.19 ± 0.89	2.31 ± 0.90	0.501
	After	0.73 ± 0.72	2.36 ± 0.93	<0.001
	*p* value^*∗∗*^	<0.001	0.161	

Weight gain	Before	1.71 ± 0.78	1.42 ± 0.90	0.074
	After	0.00 ± 0.00	1.11 ± 0.85	<0.001
	*p* value	<0.001	0.356	

Felt colder than other people	Before	1.54 ± 0.98	1.47 ± 0.98	0.729
	After	0.02 ± 0.14	1.93 ± 1.12	<0.001
	*p* value	<0.001	<0.001	

Constipation	Before	1.06 ± 0.94	0.98 ± 0.97	0.682
	After	0.38 ± 0.69	1.04 ± 1.05	<0.001
	*p* value	0.004	0.664	

Hair loss or coarseness	Before	1.63 ± 1.19	1.31 ± 1.25	0.170
	After	1.40 ± 1.19	1.33 ± 1.26	0.748
	*p* value	0.017	0.327	

Skin dryness or coarseness	Before	1.13 ± 1.03	0.89 ± 0.90	0.194
	After	1.62 ± 1.16	1.04 ± 0.98	0.006
	*p* value	<0.001	0.011	

Nail brittleness or flaking	Before	0.40 ± 0.69	0.42 ± 0.69	0.915
	After	0.40 ± 0.69	0.44 ± 0.69	0.808
	*p* value	0.999	0.999	

Loss of appetite	Before	0.13 ± 0.44	0.33 ± 1.16	0.263
	After	0.00 ± 0.00	0.33 ± 1.16	0.044
	*p* value	0.056	1.000	

Hearing problems	Before	0.48 ± 0.73	0.22 ± 0.57	0.039
	After	0.38 ± 0.66	0.22 ± 0.57	0.165
	*p* value	0.043	0.999	

Voice hoarseness or huskiness	Before	0.56 ± 0.80	0.60 ± 0.83	0.789
	After	0.42 ± 0.67	0.58 ± 0.85	0.288
	*p* value	0.011	0.327	

Speech change (slowness, inaccuracy)	Before	0.56 ± 1.51	0.22 ± 0.50	0.118
	After	0.31 ± 0.64	0.22 ± 0.50	0.421
	*p* value	0.282	0.999	

Memory problems	Before	1.63 ± 0.93	1.53 ± 0.96	0.558
	After	0.75 ± 0.76	1.60 ± 0.99	<0.001
	*p* value	<0.001	0.161	

Concentration disturbance	Before	1.29 ± 0.91	1.07 ± 0.81	0.200
	After	0.58 ± 0.72	1.05 ± 0.85	0.002
	*p* value	<0.001	1.000	

Giddy or dizzy feeling	Before	0.81 ± 0.72	0.67 ± 0.64	0.305
	After	0.10 ± 0.41	0.71 ± 0.69	<0.001
	*p* value	<0.001	0.574	

Depressed or low feeling	Before	1.75 ± 0.99	1.78 ± 1.12	0.877
	After	1.54 ± 1.06	1.82 ± 1.12	0.188
	*p* value	0.376	0.574	

ThySRQ: Thyroid Symptom Rating Questionnaire. ^*∗*^*p* value Student's *t*-test. ^*∗∗*^*p* value paired *t*-test.

**Table 3:** Comparison of changes in mean anthropometric and laboratory indices within and between intervention groups.

Parameters	Subgroups	Ginger (mean ± SD), *n* = 27	Placebo (mean ± SD), *n* = 26	*p* value independent *t*-test
Weight (kg)	Before	85.15 ± 17.95	81.69 ± 15.97	0.463
	After	82.72 ± 18.07	82.81 ± 15.90	0.986
	Change	−2.43 ± 1.23	1.12 ± 1.18	0.001
	*p* value paired *t*-test	0.001	0.001	

BMI (kg/m^2^)	Before	31.17 ± 5.28	31.29 ± 5.12	0.928
	After	30.27 ± 5.32	31.71 ± 4.99	0.315
	Change	−0.90 ± 0.46	0.42 ± 0.43	0.001
	*p* value paired *t*-test	0.001	0.001	

Waist size (cm)	Before	110.30 ± 11.52	109.5 ± 13.92	0.821
	After	106.33 ± 11.25	109.19 ± 11.41	0.363
	Change	−3.96 ± 1.81	−0.31 ± 8.97	0.043
	*p* value paired *t*-test	0.001	0.863	

TSH	Before	3.62 ± 2.21	2.87 ± 1.88	0.185
	After	2.76 ± 1.72	3.81 ± 1.95	0.042
	Change	−0.89 ± 1.33	0.95 ± 1.45	0.001
	*p* value paired t-test	0.001	0.003	

FBS	Before	99.74 ± 13.56	86.19 ± 8.32	0.001
	After	88.52 ± 10.41	93.15 ± 8.78	0.086
	Change	−11.65 ± 6.55	6.96 ± 7.51	0.001
	*p* value paired t-test	0.001	0.001	

TG	Before	229.52 ± 106.5	163.04 ± 79.49	0.013
	After	175.03 ± 80.28	203.93 ± 104.52	0.266
	Change	−56.59 ± 67.57	40.89 ± 57.44	0.001
	*p* value paired t-test	0.001	0.001	

TChol	Before	181.56 ± 41.54	170.88 ± 37.11	0.329
	After	162.89 ± 32.87	181.27 ± 37.21	0.062
	Change	−19.38 ± 24.02	10.38 ± 16.66	0.001
	*p* value paired t-test	0.001	0.004	

IPAQ	Before	200.36 ± 95.69	200.35 ± 61.44	1.000
	After	200.36 ± 95.28	200.28 ± 60.46	0.997
	*p* value paired *t*-test	0.995	0.915	

BMI: body mass index; TSH: thyroid-stimulating hormone; FBS: fasting blood sugar; TG: triglyceride; TChol: total cholesterol; IPAQ: International Physical Activity Questionnaire.

**Table 4:** The results of gender-based subgroup analysis showing changes in IPAQ domains and laboratory indices in males and females who received ginger.

IPAQ domains and laboratory indices	Male	Female	*P* value
Tiredness	−1.33 ± 0.71	−1.72 ± 0.57	0.137
Weight gain	−1.67 ± 0.87	−1.72 ± 0.83	0.873
Felt colder than other people	−1.89 ± 0.93	−1.72 ± 0.96	0.671
Constipation	0.00 ± 1.32	−0.94 ± 0.73	0.053
Hair loss or coarseness	−0.22 ± 0.44	−0.28 ± 0.57	0.801
Skin dryness or coarseness	0.67 ± 0.50	0.44 ± 0.51	0.294
Nail brittleness or flaking	0.00 ± .00	0.00 ± .00	0.99
Loss of appetite	−0.11 ± 0.33	−0.28 ± 0.67	0.49
Hearing problems	−0.11 ± 0.33	−0.17 ± 0.38	0.715
Voice hoarseness or huskiness	0.00 ± 0.00	−0.33 ± 0.49	0.052
Speech change (slowness, inaccuracy)	0.00 ± 0.00	−0.61 ± 2.35	0.448
Memory problems	−0.67 ± 0.50	−1.11 ± 0.58	0.062
Concentration disturbance	−0.67 ± 0.50	−0.94 ± 0.42	0.139
Giddy or dizzy feeling	−0.78 ± 0.67	−0.94 ± 0.64	0.534
Depressed or low feeling	0.11 ± 0.33	−0.22 ± 0.73	0.209
TSH	−0.75 ± 0.69	−0.97 ± 1.59	0.702
FBS	−14.33 ± 3.94	−10.24 ± 7.28	0.132
TG	−51.44 ± 48.36	−59.31 ± 77.08	0.784
TChol	−20.33 ± 18.63	−18.88 ± 26.96	0.887

IPAQ: International Physical Activity Questionnaire; FBS: fasting blood sugar; TG: triglyceride; TChol: total cholesterol. Negative values show a decrease in the mean scores through the intervention.

## Data Availability

The data used to support the findings of this study are available on request from the corresponding author.
